# Meta-Analysis of Lung Cancer Risk from Exposure to Diesel Exhaust: Vermeulen et al. Respond

**DOI:** 10.1289/ehp.1408428R

**Published:** 2014-09-01

**Authors:** Roel Vermeulen, Lutzen Portengen, Debra T. Silverman, Eric Garshick, Kyle Steenland

**Affiliations:** 1 Utrecht University, Utrecht, the Netherlands; 2 National Cancer Institute, Rockville, Maryland, USA; 3 VA Boston Healthcare System, Boston, Massachusetts, USA; 4 Emory University, Atlanta, Georgia, USA

Crump asserts that we “inappropriately mixed data from exposures lagged 5 years and 15 years” in our study published in *Environmental Health Perspectives* (Vermeulen et al. 2014). Exposure metrics from different studies available for meta-analysis are rarely, if ever, completely comparable. Therefore, even exposures labeled exactly the same (e.g., “cumulative exposure lagged 15 years”) differ between studies because of differences in exposure assessment (cumulative exposure is accrued in different ways in different populations) and because of differences in age composition and extent of follow-up. As a result, in epidemiological studies, optimal lag times to exclude exposures not affecting risk may be variable across studies. For our meta-regression, we included the exposure–response relationships that were presented as the main analyses in the respective papers. For the two trucking studies (Garshick et al. 2012; Steenland et al. 1998), the exposure metric was lagged 5 years, whereas for the Diesel Exhaust in Miners Study (DEMS; Silverman et al. 2012) it was 15 years. Results for an analysis using a 5-year lag in DEMS were not published, although the 5-year lag had been included in analyses to examine changes in model fit as a function of lag. From those analyses (Silverman et al. 2012, Supplementary Figure 1), it is apparent that a 5-year lag showed the worst model fit of all lags (0–25 years); thus, it does not make sense to use this particular analysis as the primary exposure–response relation simply because the label “5-year lag” coincides with that of the other two studies.

We acknowledge that the interpretation of the risk function may be affected by differences in exposure between lag times. For this reason, we performed sensitivity analyses that included different lags from each study; overall results changed only slightly. We extended our earlier sensitivity analyses by including the unpublished 5-year lagged data from Silverman et al. (Silverman DT, personal communication).We note again, however, that these 5-year lagged data from the DEMS do not fit nearly as well as the 15-year lagged data. We stress our belief that they should not be the primary data for use in any risk assessment/meta-regression.

**Figure 1:**
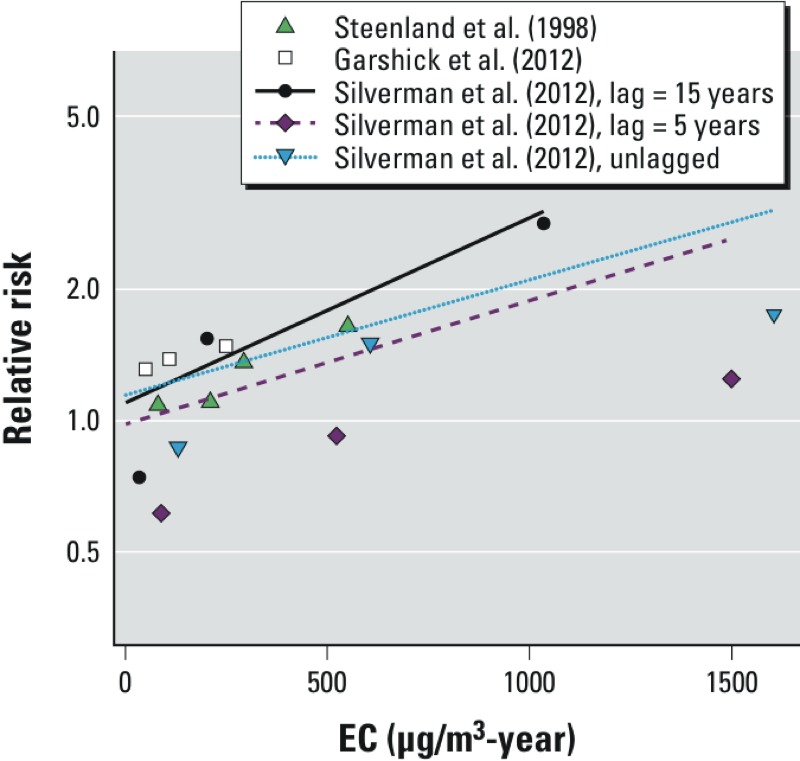
Predicted exposure–response curve of cumulative elemental carbon (EC) and lung cancer risk using different lag-times based on a log-linear regression model using relative risk estimates from the three cohort studies.

Crump argues that his alternative analyses using the 5-year lagged data were more consistent with the underlying DEMS data. Figure 1 includes the individual risk estimates for all three studies, with three alternative lag times for DEMS. It is clear from Figure 1 that the 5-year lagged risk estimates for DEMS selected by Crump for his meta-analysis are considerably lower than those of the two trucking studies and the alternative lag times for DEMS. We also included in the figure three different regression lines based on the two trucking studies and one of three different sets of results for DEMS, obtained using the exposure data lagged 0, 5, and 15 years. All models are fitted using the full estimated covariance matrix to appropriately account for the correlation between categorical point estimates from the same study, a correction ignored by Crump. The lowest meta-regression slope, using the 5-year lagged exposure results from DEMS, *a*) is higher than that reported by Crump using the variance estimates only; *b*) is statistically significant (0.00065; 95% CI: 0.00028, 0.0010); and *c*) falls within our previous sensitivity analyses (Vermeulen et al. 2014). It is also clear that the exposure–response function derived using the 15-year lagged exposure data from DEMS is a much better fit overall.

In summary, we strongly disagree with the assertions made by Crump in his letter that we inappropriately mixed lag times. On his other points, we note that adjustment for employment duration in the trucking industry cohort study was not a second exposure measure, but appropriately reduced bias attributable to a healthy worker survivor effect.

Additional analyses presented here confirm that the original findings from the meta-analyses are robust. Therefore, we firmly stand with the conclusions of our original paper.
